# Chromosome congression is promoted by CENP-Q- and CENP-E-dependent pathways

**DOI:** 10.1242/jcs.163659

**Published:** 2015-01-01

**Authors:** James Bancroft, Philip Auckland, Catarina P. Samora, Andrew D. McAinsh

**Affiliations:** Mechanochemical Cell Biology Building, Division of Biomedical Cell Biology, Warwick Medical School, University of Warwick, Coventry CV4 7AL, UK

**Keywords:** CENP-E, CENP-Q, Congression, Kinetochore, Mitosis

## Abstract

A key step of mitosis is the congression of chromosomes to the spindle equator. Congression is driven by at least two distinct mechanisms: (1) kinetochores slide along the microtubule lattice using the plus-end directed CENP-E motor, and (2) kinetochores biorientating near the pole move to the equator through microtubule depolymerisation-coupled pulling. Here, we show that CENP-Q – a subunit of the CENP-O complex (comprising CENP-O, CENP-P, CENP-Q and CENP-U) that targets polo-like kinase (Plk1) to kinetochores – is also required for the recruitment of CENP-E to kinetochores. We further reveal a CENP-E recruitment-independent role for CENP-Q in depolymerisation-coupled pulling. Both of these functions are abolished by a single point mutation in CENP-Q (S50A) – a residue that is phosphorylated *in vivo*. Importantly, the S50A mutant does not affect the loading of Plk1 onto kinetochores and leaves the CENP-O complex intact. Thus, the functions of CENP-Q in CENP-E loading and depolymerisation-coupled pulling are independent from its role in Plk1 recruitment and CENP-O complex stabilisation. Taken together, our data provide evidence that phosphoregulation of CENP-Q plays a central function in coordinating chromosome congression mechanisms.

## INTRODUCTION

Chromosome congression is the process by which chromosomes align at the spindle equator, a position equidistant from both spindle poles (Kops et al., 2010; Walczak et al., 2010). In human cells, this process takes ∼15-20 min and results in the formation of the metaphase plate. Chromosomes remain in metaphase for a similar period of time before sister chromatids are segregated to opposite spindle poles in anaphase. Congression is coincident with the process of chromosome biorientation, that is the establishment of amphitelic (or bipolar) attachments in which sister kinetochores attach to microtubules emanating from opposite spindle poles. Such amphitelic attachments are compatible with oscillatory motion because sisters switch between poleward and away from pole movement states (Skibbens et al., 1993). These chromosomes are able to congress because they undergo sustained movements towards the spindle equator. The force for these movements comes from the poleward-moving sister kinetochore remaining attached to depolymerising kinetochore–microtubule plus-ends (from here on called depolymerisation-coupled pulling) (Coue et al., 1991; Koshland et al., 1988; Mitchison and Salmon, 1992). Congression can also take place before biorientation: mono-orientated kinetochores have been shown to engage with the lattice of pre-existing kinetochore–microtubule fibres (K-fibres) and other stabilized microtubule bundles through the second (leading) sister, and they then slide towards the spindle equator (Kapoor et al., 2006; Cai et al., 2009). Once at the metaphase plate, these kinetochores then biorientate. Furthermore, work in human cells and mouse oocytes reveals that chromosomes are arranged in a ring-like conformation early in prometaphase that favours the ‘instantaneous’ biorientation of sister kinetochores at the spindle equator (Kitajima et al., 2011; Magidson et al., 2011). Thus, cells have multiple mechanisms to position chromosomes on the metaphase plate.

The key challenge is to identify the molecules that are involved in these congression mechanisms. The sliding of chromosomes by laterally attached kinetochores is mediated by the minus-end-directed motor dynein (Yang et al., 2007) or the plus-end-directed kinesin CENP-E (Kapoor et al., 2006; Kim et al., 2010). Following depletion or inhibition of CENP-E, the majority of chromosomes congress, forming a metaphase plate; however, a subset remain trapped around the spindle poles – these are termed polar chromosomes (Putkey et al., 2002). In contrast to these lateral-sliding mechanisms, the molecules required for depolymerisation-coupled pulling are largely unknown. Such a mechanism must involve proteins that (1) mediate end-on microtubule–kinetochore attachments, (2) maintain attachment to depolymerising microtubules and (3) ensure the K-fibre remains in a net depolymerising state through control of kinetochore–microtuble dynamics. The Ndc80 and Ska complexes can directly bind to microtubules *in vitro*, with the Ska complex (but not the Ndc80 complex) able to autonomously track depolymerising microtubule plus-ends (Schmidt et al., 2012). Extensive depletion of either complex results in a severe failure in chromosome congression and the inability to form a metaphase plate (Daum et al., 2009; Gaitanos et al., 2009; Welburn et al., 2010). Thus, the Ndc80 and Ska complexes are proposed to form the end-on microtubule attachment sites within the kinetochore.

Whether the process of end-on attachment can be separated from the mechanism of depolymerisation-coupled pulling is unclear. In this regard, our previous work has shown that the constitutive centromere-associated network (CCAN) is required for normal chromosome congression, but that it is not required for the formation of end-on attachments (Amaro et al., 2010; McClelland et al., 2007; Toso et al., 2009). The CCAN is a 17-subunit complex that contains a set of core factors that directly bind to CENP-A (CENP-C and CENP-N) and to Histone 3 (H3) nucleosomes (CENP-T, CENP-W, CENP-S and CENP-X) (for reviews, see Perpelescu and Fukagawa, 2011; McAinsh and Meraldi, 2011; Westhorpe and Straight, 2013). The core CCAN recruits the extended CCAN, which includes the complex comprising CENP-H, CENP-I, CENP-K and CENP-M (Basilico et al., 2014), and the CENP-O complex, which comprises CENP-O, CENP-P, CENP-Q and CENP-U (Hori et al., 2008). However, the function of this extended CCAN is poorly understood. Phenotypic analysis shows that depletion of such CCAN subunits can interfere with timely chromosome congression, as well as altering kinetochore–microtubule plus-end turnover, K-fibre stability and poleward microtubule flux (Amaro et al., 2010; Foltz et al., 2006; Hori et al., 2008; McClelland et al., 2007; Mchedlishvili et al., 2012). The CENP-O complex also plays a role in the recruitment of other key kinetochore components, including Plk1 through interaction with phosphorylated residue T78 of CENP-U (Kang et al., 2011). Moreover, the CENP-Q and CENP-U subunits bind directly to taxol-stabilised microtubules *in vitro* (Amaro et al., 2010; Hua et al., 2011), suggesting that they might have direct roles in regulating the kinetochore microtubule and, potentially, depolymerisation-coupled pulling.

## RESULTS

### Depletion of CENP-Q causes accumulation of polar chromosomes

**Fig. 1.**
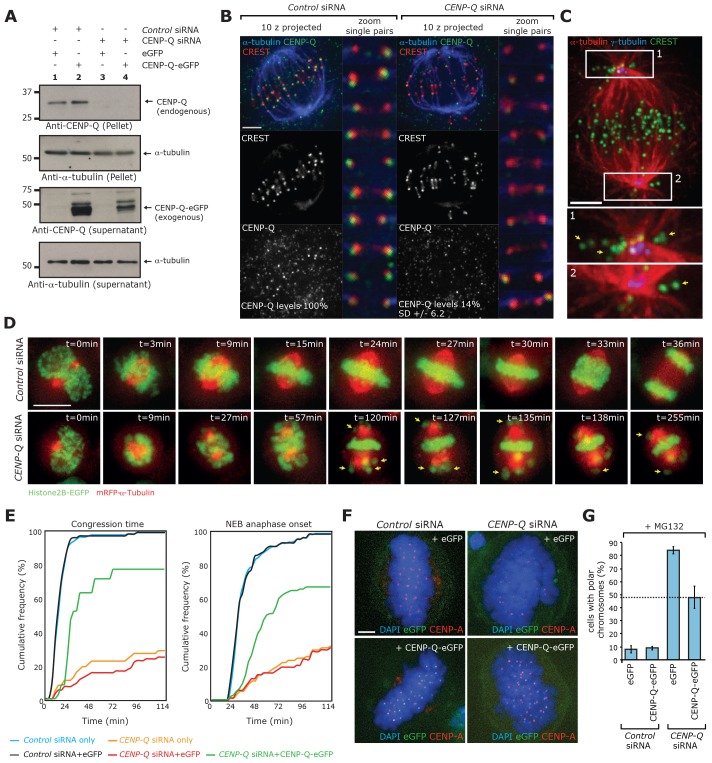
**Depletion of the outer-plate protein CENP-Q causes accumulation of polar chromosomes.** (A) Immunoblots of whole-cell HeLa E1 lysates that had been transfected with control siRNA or an siRNA against CENP-Q (CENP-Q siRNA) for 12 h and then transfected with either empty vector or an siRNA-resistant vector expressing CENP-Q tagged with eGFP for 48 h. The blot was probed with antibodies against CENP-Q and α-tubulin. Endogenous CENP-Q and the corresponding α-tubulin control are shown in the top two panels. The pellet fraction is shown as nonspecific staining obscuring the protein in the supernatant. Expression of the transgene and corresponding α-tubulin loading control are shown in the lower two panels. (B) Immunofluorescence microscopy images of metaphase cells and magnified (zoom) kinetochore pairs in cells transfected with a control siRNA or CENP-Q siRNA that were then stained with CREST antisera (red) and antibodies against CENP-Q (green) and α-tubulin (blue). The images of metaphase cells correspond to *z*-projections (10 focal planes at 0.2 µm spacing) and the zoom images of the single kinetochores are from a single focal plane of the stack. Values at the base of the bottom two panels correspond to the relative values of CENP-Q staining in control and CENP-Q-depleted cells (*n* = 3, 150 kinetochores, 30 cells). (C) Immunofluorescence microscopy images of a CENP-Q-depleted metaphase HeLa E1 cell stained with CREST antisera (green) and an antibody against α-tubulin (red). The image is a *z*-projection (10 focal planes at 0.2 µm spacing). Zoom boxes 1 and 2 are centred on the spindle poles, and yellow arrows point to unaligned kinetochores around the spindle poles. (D) Frames from live-cell movies of HeLa E1 cells co-expressing H2B–eGFP and mRFP–α-tubulin in control (top row) and CENP-Q-siRNA-treated cells (second row). Yellow arrows point to unaligned kinetochores. Videos of control and CENP-Q-depleted cells are available in in supplementary material Movies 1 and 2, respectively. t = 0, point of nuclear envelope breakdown. (E) Quantification of the time from nuclear envelope breakdown (NEB) to the time when the last chromosome congressed to the metaphase plate, and NEB to the time of anaphase onset. Blue and orange lines indicate the timings of HeLa E1 cells co-expressing H2B–eGFP and mRFP–α-tubulin in control and CENP-Q-siRNA-treated cells, respectively. Black green and red lines represent timing data from cells expressing H2B–mRFP that had been treated with control siRNA and siRNA-resistant CENP-Q–eGFP vector (black), CENP-Q siRNA and siRNA-resistant CENP-Q–eGFP vector (green) and CENP-Q siRNA and eGFP vector (red). (F) Immunofluorescence microscopy images (*z*-projections, 10 focal planes at 0.2 µm spacing) of a CENP-Q-siRNA rescue experiment in HeLa E1 cells. Cells were treated with CENP-Q siRNA or control siRNA for 14 h and then transfected with an siRNA-resistant CENP-Q–eGFP construct or a control eGFP expression plasmid for a further 48 h. To reduce the effect of the mitotic stage on alignment, cells were treated with 1 µM MG132 for 90 min before fixation. Cells were stained with DAPI and probed with an antibody against CENP-A. (G) Quantification of cells with polar chromosomes in the rescue experiment described in F. *n* = 2, ≥100 poles, 50 cells. Scale bars: 3 µm (B,C,F); 10 µm (D).

To investigate the function of human CENP-Q, we depleted the protein in HeLa cells using small interfering RNA (siRNA) oligonucleotides. Immunoblotting with antibodies against CENP-Q demonstrated that the total protein levels were reduced to non-detectable levels (Fig. 1A). Moreover, quantitative immunofluorescence confirmed that the kinetochore-bound population of the CENP-Q protein was reduced by 86% (±6.2) (relative to CENP-A) following siRNA-mediated depletion (Fig. 1B). Previous work (Hori et al., 2008; Kang et al., 2006) has demonstrated that depletion of CENP-Q destabilises the binding of other CENP-O complex subunits to kinetochores. We were able to confirm these observations as depletion of CENP-Q resulted in the loss of CENP-O from kinetochores (supplementary material Fig. S1A). Moreover, depletion of CENP-Q reduced the levels of Plk1 at kinetochores by 84% (supplementary material Fig. S1B,C, *n* = 3, 300 kinetochores, 30 cells). Consistent with a kinetochore-specific effect, CENP-Q depletion did not affect the polar localisation of Plk1 (supplementary material Fig. S1B,C, *n* = 3, 120 poles, 60 cells). To rule out off target effects, we performed a rescue experiment where cells treated with a control or CENP-Q siRNA were transfected with an siRNA-resistant enhanced green fluorescent protein eGFP tagged CENP-Q (CENP-Q-eGFP) or an empty vector. The transgene partially rescued kinetochore-bound levels of Plk1 to 50% of those observed in control-siRNA-treated cells that had been transfected with the empty vector (supplementary material Fig. S1B,C, *n* = 3, 600 kinetochores, 60 cells). Immunoblotting confirmed that the siRNA against CENP-Q depleted the endogenous protein without affecting the expression of the CENP-Q-eGFP transgene used for these rescue experiments (Fig. 1A). These data confirm that the CENP-O complex is a key platform for the recruitment of Plk1 to kinetochores.

Depletion of CENP-Q did not, however, affect the kinetochore binding of Ndc80, Kif18A, Ska3 or MCAK (supplementary material Fig. S1A), indicating that CENP-Q does not play a major role in global kinetochore architecture. This conclusion is consistent with the ability of CENP-Q-depleted cells to form a normal metaphase plate (Fig. 1C,D). We did, however, note that the loss of CENP-Q from kinetochores had a striking effect on the position of a subset of chromosomes within the spindle; a number of kinetochores were trapped around the spindle poles (from here on termed polar chromosomes; Fig. 1C). This phenotype can also be observed in images of CENP-Q-knockout DT40 cells (Hori et al., 2008) or CENP-U-depleted human cells (Kang et al., 2006). To further assess this phenotype, we collected time-lapse movies in living HeLa cells co-expressing Histone2B–eGFP and monomer red fluorescent protein (mRFP)-tagged α-tubulin (mRFP–α-tubulin, Fig. 1D; supplementary material Movies 1, 2). Taking T = 0 to be the point of nuclear envelope breakdown, we determined the timing of chromosome congression. Only 10.3% of CENP-Q-depleted cells had formed a metaphase plate and aligned all their chromosomes by T = 24 min, compared with >95% in control cells (Fig. 1D,E). Consistent with our fixed-cell experiments, a number of chromosomes remained trapped around the spindle pole (yellow arrows in Fig. 1D). These chromosome congression defects were associated with a prolonged mitotic delay, with only 26% of CENP-Q-depleted cells undergoing anaphase within 96 min, compared with 99% of control cells (Fig. 1E). Of the cells that underwent anaphase within 114 min, 19% failed to congress all chromosomes. This is consistent with previous work showing that the knockout of CENP-Q in chicken DT40 cells causes a mitotic delay and congression defects (Hori et al., 2008).

To rule out off-target effects, we depleted cells of CENP-Q using siRNA and transfected them with either an siRNA-resistant CENP-Q–eGFP transgene or an empty vector. We next treated the cells with MG132 for 90 min (to prevent anaphase onset and therefore rule out differences associated with variable cell cycle stage) (Klebig et al., 2009) before fixation and staining with antibodies against CENP-A (to mark kinetochores) and DAPI (to mark the chromosome position). This allowed the proportion of cells with polar chromosomes to be calculated. Depletion of CENP-Q and transfection with empty vector resulted in 84% (±3.5%) of metaphase cells having unaligned kinetochores that were situated at the pole. In contrast, transfection with an siRNA-resistant CENP-Q transgene greatly reduced the proportion of cells with polar chromosomes to 48% (±8%) (Fig. 1F,G). This reduction in polar chromosomes following transfection with the CENP-Q transgene also resulted in an increased rate of chromosome congression (compared with that of vector alone), with approximately twice the number of cells entering anaphase when assessed by using live-cell imaging of Histone-2B–GFP-expressing cells (Fig. 1E). Finally, a second siRNA that targets CENP-Q gave the same polar chromosome and congression phenotype (supplementary material Fig. S2A–C), as did depletion of the CCAN subunit CENP-P, which has been shown to recruit CENP-Q to kinetochores (supplementary material Fig. S2A–C) (Foltz et al., 2006).

Taken together, these data show that kinetochores lacking CENP-Q (and thus the CENP-O complex) are defective in the timely congression of most chromosomes and that they are also unable to transport a subset of chromosomes that are positioned near the spindle pole to the metaphase plate.

### CENP-Q is required to load CENP-E onto kinetochores

**Fig. 2.**
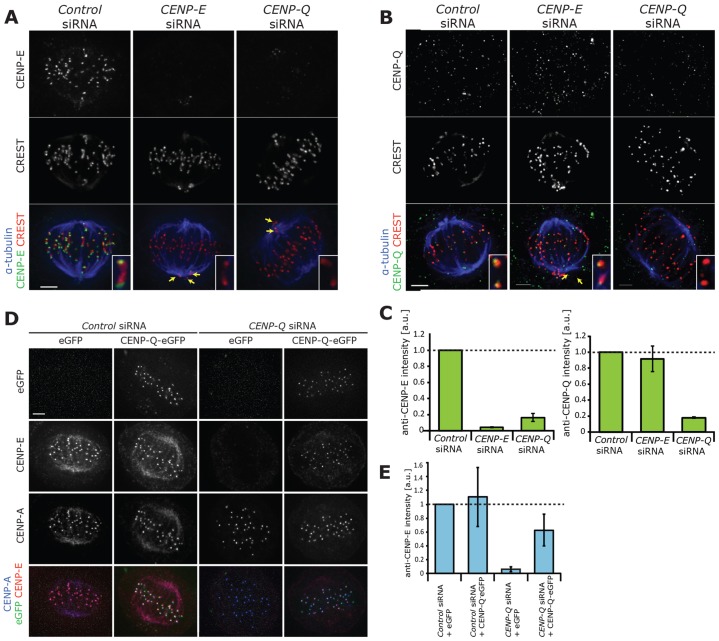
**CENP-Q is required to load CENP-E to kinetochores.** (A) Immunofluorescence microscopy images of a metaphase HeLa E1 cells treated with control siRNA, or siRNA against CENP-E (CENP-E siRNA) or CENP-Q for 48 h and then stained with CREST antisera (red) and antibodies against CENP-E (green) and α-tubulin (blue). The image is a *z*-projection (10 focal planes at 0.2 µm spacing). Yellow arrows point to unaligned kinetochores. Insets show single kinetochore pairs. (B) Immunofluorescence microscopy images of metaphase HeLa E1 cells treated with control, CENP-E or CENP-Q siRNA for 48 h and stained with CREST antisera (red) and antibodies against CENP-Q (green) and α-tubulin (blue). The image is a *z*-projection (10 focal planes at 0.2 µm spacing). Yellow arrows point to unaligned kinetochores. Insets show single kinetochore pairs. (C) Left panel, quantification of CENP-E immunofluorescence levels in HeLa E1 cells treated with control siRNA, CENP-E siRNA or CENP-Q siRNA for 48 h. The intensities are determined at each kinetochore relative to that of CREST after background subtraction, *n*≥150 kinetochores per condition from three independent experiments. Dashed line indicates CENP-E levels in control siRNA cells. Error bars indicate ±s.d. Right panel, quantification of CENP-Q immunofluorescence levels in HeLa E1 cells treated with control siRNA, CENP-E siRNA or CENP-Q siRNA for 48 h. Intensities are determined at each kinetochore relative to that of CREST after background subtraction, *n*≥100 kinetochores per condition from two independent experiments. Dashed line indicates CENP-Q levels in control cells. Error bars indicate ±s.d. (D) Immunofluorescence microscopy images (*z*-projections, 10 focal planes at 0.2 µm spacing) of a CENP-Q siRNA rescue experiment in HeLa E1 cells. Cells were treated with CENP-Q siRNA or control siRNA for 12 h and then transfected with an siRNA-resistant plasmid expressing CENP-Q–eGFP or a control eGFP expression plasmid for a further 48 h. To reduce the effect of the mitotic stage on alignment, cells were arrested in metaphase with MG132 at a 1 µM final concentration for 90 min, followed by fixation. Cells were stained with antibodies against CENP-A (blue) and CENP-E (red). (E) Quantification of CENP-E immunofluorescence levels in the CENP-Q siRNA rescue experiment detailed in D. Intensities were determined at each kinetochore relative to that of CENP-A after background subtraction, *n*≥150 kinetochores per condition from three independent experiments. Dashed line indicates CENP-Q levels in cells treated with the control siRNA. Error bars indicate ±s.d. Scale bars: 3 µm (A,B,D).

The polar chromosome phenotype in CENP-Q-depleted cells is very reminiscent of that reported for the depletion of the kinesin 7 family member CENP-E in human cells (Putkey et al., 2002; Weaver et al., 2003). We therefore investigated whether CENP-Q was required for the loading of CENP-E onto kinetochores. We confirmed with immunofluorescence that the treatment of cells with an siRNA targeting CENP-E reduced the levels of the motor protein on kinetochores by 95.6% (Fig. 2A,C, 150 kinetochores). Depletion of CENP-Q also reduced the levels of CENP-E on kinetochores by 80% (Fig. 2A,C; 100 kinetochores). Thus, phenotypes associated with CENP-Q depletion must be considered as a composite of losing both CENP-Q and CENP-E. We next performed the reciprocal experiment and found that depletion of CENP-E did not affect binding of CENP-Q to kinetochores (Fig. 2B,C). To confirm these results, we treated cells with control or CENP-Q siRNA and then transfected with either the siRNA-resistant CENP-Q–eGFP transgene or an empty vector and quantified the levels of CENP-E on kinetochores. Transfection with the transgene partially rescued the binding to kinetochores of CENP-E to ∼50% of the intensity measured in control-siRNA-treated cells that had been transfected with the empty vector (Fig. 2D,E). These data indicate that CENP-Q is required to load CENP-E onto kinetochores.

### Fate of unaligned kinetochore pairs in CENP-Q- and CENP-E-depleted cells

**Fig. 3.**
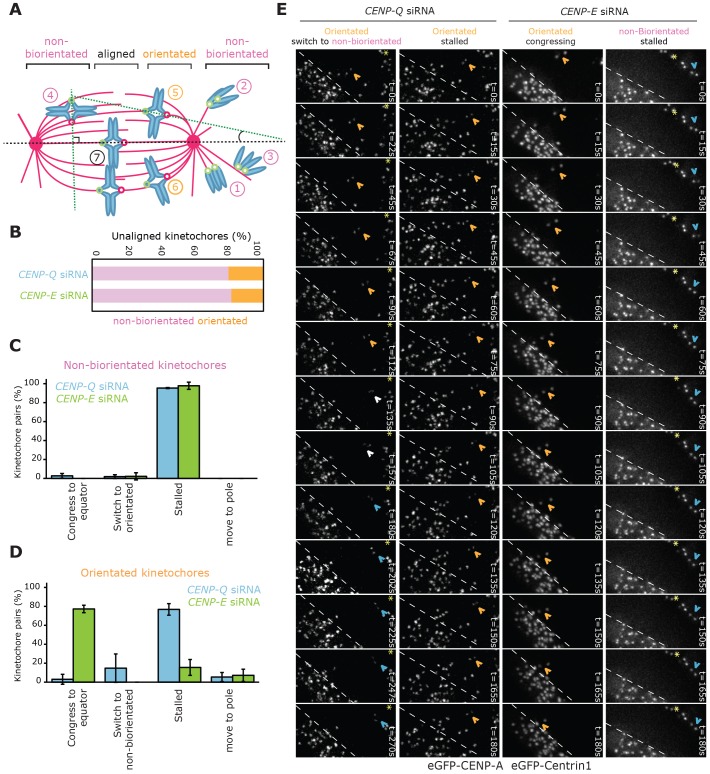
**Fates of unaligned kinetochore pairs.** (A) Schematic representing the orientation of unaligned kinetochore pairs within the mitotic spindle. The black dotted line represents the pole-to-pole axis, dotted green lines on chromosomes 4 and 5 represent the kinetochore sister–sister axis, the ∼90° angle between the sister–sister axis of chromosome 4 and the spindle pole-to-pole axis indicates non-biorientation. The reduced angle (≤45°) of the sister–sister axis relative to spindle axis of chromosome 5 indicates biorientation. Chromosomes 1, 2 and 3 are behind the pole and are therefore non-biorientated. Chromosome 6 is mono-orientated by the pole proximal kinetochore and laterally attached to an adjacent K-fibre by the pole distal kinetochore. Discriminating this chromosome from biorientated chromosomes (e.g. number 5) was not possible in our assay. (B) The orientation state of unaligned kinetochore pairs in CENP-Q- (*n* = 217 kinetochores) and CENP-E-siRNA- (*n* = 211 kinetochores) treated HeLa K cells stably expressing eGFP–CENP-A and eGFP–centrin1. Pink represents non-biorientated kinetochores (classes 1, 2, 3 and 4), and orange orientated kinetochores (classes 5 and 6). *n*≥3 independent experiments. (C) The fates of non-biorientated kinetochore pairs over 5 min in live HeLa K cells (stably expressing eGFP–CENP-A and eGFP–centrin1) after 48 h of treatment with CENP-Q or CENP-E siRNA. (D) The fates of orientated kinetochore pairs over 5 min in live HeLa K cells (stably expressing eGFP–CENP-A and eGFP–centrin1) after 48 h of treatment with CENP-Q or CENP-E siRNA. (E) Example frames from movies of kinetochore fates in HeLa K cells (stably expressing eGFP–CENP-A and eGFP–centrin1) after 48 h of treatment with CENP-Q or CENP-E siRNA. Orange chevrons indicate pairs that are orientated with the spindle axis, blue chevrons indicate pairs that are non-biorientated and white chevrons indicate where the designation of state is unclear. Yellow stars indicate the spindle pole when visible. The first column shows an example of a biorientated kinetochore pair in a CENP-Q-depleted cell, the pair does not congress but switches to a non-biorientated state (supplementary material Movie 3). The second column shows an example of an orientated kinetochore pair that fails to congress in CENP-Q-depleted cells (supplementary material Movie 4). In contrast, the third column shows an example of an orientated sister pair in CENP-E-depleted cells that is still able to congress to the metaphase plate (supplementary material Movie 5). The forth column shows an unaligned non-biorientated sister pair that cannot move to the metaphase plate in a CENP-E-depleted cell (supplementary material Movie 6). t = 0, first frame of the movie. The dashed lines indicate the metaphase plate periphery.

The key question is whether the polar chromosome phenotype in CENP-Q-depleted cells simply reflects the unbinding of CENP-E motor proteins from kinetochores, or whether CENP-Q makes additional contributions to chromosome congression that are independent of CENP-E recruitment. We reported above, we were in a position to answer this question because depletion of CENP-E did not affect CENP-Q binding to kinetochores, compared with depletion of CENP-Q that also removed CENP-E. We therefore determined how these differing kinetochore states affected the congression of chromosomes by tracking the fates of uncongressed kinetochore pairs by using live-cell microscopy. For this, we used HeLa K cells expressing eGFP–CENP-A (a kinetochore marker) and eGFP–centrin1 (a spindle pole marker), and collected time-lapse movies over the course of 5 min in both CENP-Q-depleted cells and CENP-E-depleted cells. Sister pairs were assigned as non-biorientated (as judged in in the first frame of the movie; supplementary material Movies 3–6) if the sister–sister axis was rotated by approximately 90° relative to the pole-to-pole axis (see schematic in Fig. 3A). Biorientation would be improbable with this geometry because kinetochores could not make end-on attachments with microtubules coming from opposite spindle poles. Similarly, kinetochore pairs positioned behind the spindle pole were classed as non-biorientated because, in this state, biorientation would be geometrically impossible. In contrast, kinetochore pairs with a sister–sister axis of ∼45° or less relative to the pole–pole axis were classified as orientated. We cannot be sure that these sisters are biorientated because it is not possible to distinguish this state from sister pairs in which one sister is mono-orientated and the second is laterally attached (Kapoor et al., 2006; schematic in Fig. 3A). The relative proportion of orientated and non-biorientated uncongressed kinetochore pairs was very similar in CENP-Q- and CENP-E-depleted cells, with around 80% occupying a non-biorientated state (Fig. 3B). The fates of these non-biorientated unaligned kinetochore pairs were also very similar, with the majority (>90%) remaining stalled and unable to progress towards the spindle equator (Fig. 3C,E).

In CENP-Q-depleted cells (where CENP-E is also absent from kinetochores), the orientated kinetochore pairs were also unable to congress and remained stalled (Fig. 3D,E; also see supplementary material Movie 4 for a further example). Surprisingly, in CENP-E-depleted cells (where CENP-Q remains bound to kinetochores), 80% of orientated sister pairs were able to congress to the metaphase plate (Fig. 3D,E; also see supplementary material Movie 5 for a further example). Because CENP-E is essential for lateral sliding, the congression events observed in CENP-E-depleted cells are almost certainly due to biorientated kinetochores utilising depolymerisation-coupled pulling.

We note that the level of kinetochore-bound CENP-E was significantly less following depletion of CENP-E compared with that upon depletion of CENP-Q (Student's *t*-test *P* = 0.0148; Fig. 2A–C). Thus, the stronger phenotype in CENP-Q-depleted cells cannot be attributed to a more efficient loss of CENP-E from kinetochores. Moreover, inhibition of CENP-E with 300 nM GSK925293, which puts the motor into a rigor state (Wood et al., 2010), gave a similar result, with almost 100% of non-biorientated kinetochores remaining stalled (supplementary material Fig. S2D) and over 50% of orientated kinetochores able to congress (supplementary material Fig. S2E). One prediction from these data is that CENP-E-depleted cells should have a higher proportion of their unaligned kinetochores trapped behind spindle poles. Indeed, 69.7% of unaligned kinetochores were behind the pole in CENP-E-depleted cells (chromosomes 1, 2 and 3 in Fig. 3A), compared with only 50.4% in CENP-Q-depleted cells. Overall, these data suggest that a CENP-Q-dependent (CENP-E-independent) mechanism is required for generating sustained plateward movement through depolymerisation-coupled pulling in order to move kinetochore pairs that reside between the pole and equator to the metaphase plate.

### CENP-Q and CENP-E generate counter forces on polar chromosomes

**Fig. 4.**
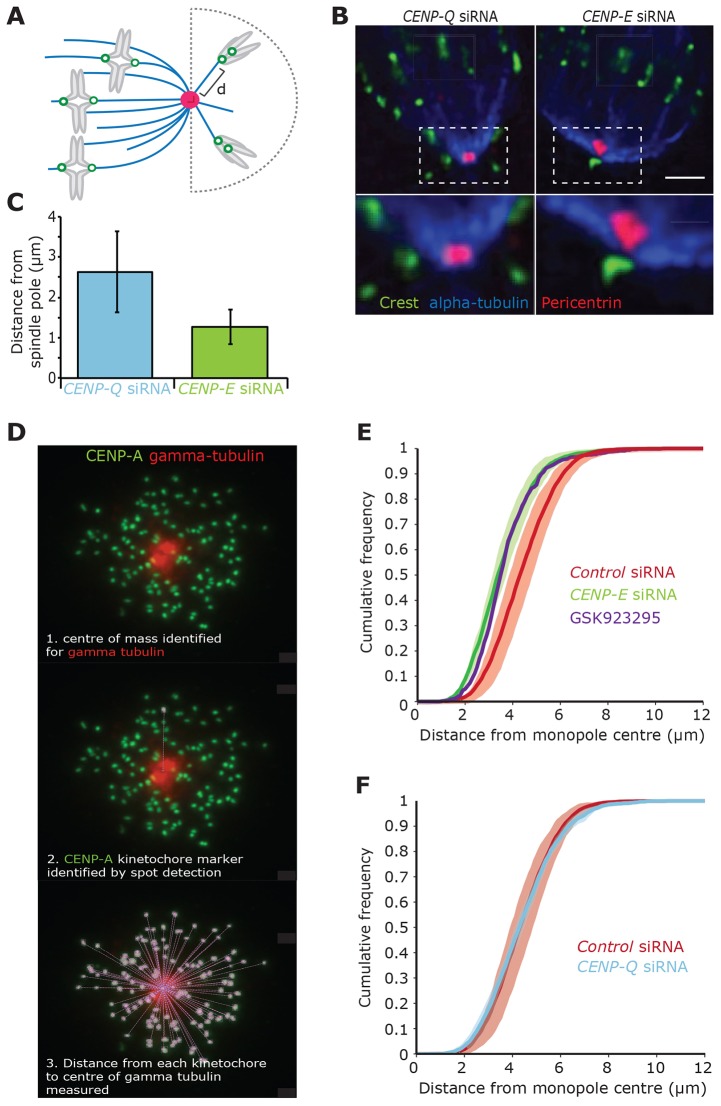
**CENP-Q and CENP-E generate counter forces on polar chromosomes.** (A) Schematic representing the measurement of the pole-to-kinetochore distances ‘d’ during depletion of CENP-Q and CENP-E. (B) Immunofluorescence microscopy images of HeLa K cells that had been treated for 48 h with CENP-Q or CENP-E siRNA and stained with CREST antisera (green) and antibodies against α-tubulin (blue) and pericentrin (red). The image is a single *z*-slice. Insets show enlarged images of the boxed areas. Scale bar: 5 µm. (C) Quantification of pole-to-kinetochore distances in CENP-Q-siRNA- (blue *n* = 2 159 kinetochores) and CENP-E-siRNA-treated (green, *n* = 2, 107 kinetochores) cells. Error bars represent ±s.d. (D) Immunofluorescence microscopy image of a HeLa E1 cell stained with antibodies against CENP-A (green) and γ-tubulin (red). Cells were treated for 48 h with control, CENP-Q or CENP-E siRNA followed by 90 min of treatment with 1 µM monastrol to induce monopolarity. The image is a *z*-projection (all focal planes, 15 µm at 0.2 µm spacing). The successive analysis overlays show the automated process of pole identification and kinetochore identification followed by distance measurement from each kinetochore to the pole. (E) Cumulative distribution of kinetochore distances from the monopole centre in cells that had been treated with control siRNA (red lines, *n* = 5 experiments with ≥8 cells), CENP-E siRNA (red lines, *n* = 4, with ≥8 cells) and GSK923295 (purple line, *n* = 1, 10 cells). Shaded areas represent ±s.d. (F) The cumulative distribution of kinetochore distances from the monopole centre in cells that had been treated with control siRNA (green lines, *n* = 5, with ≥8 cells) and CENP-Q siRNA (red lines, *n* = 3, with ≥8 cells). Shaded areas represent ±s.d.

Behind the spindle pole, the geometry of microtubules is reversed, i.e. the plus-ends of astral microtubules are proximal to the cell cortex, whereas K-fibres have plus-ends proximal to the metaphase plate. If CENP-Q directs depolymerisation-coupled pulling, it would move chromosomes poleward, whereas the CENP-E-driven lateral sliding would move them towards the plus-end (anti-poleward). Thus, we would predict that depletion of each protein would alter the positions of kinetochores located behind the pole relative to the spindle pole. To test this hypothesis, we used fixed-cell high-resolution wide-field fluorescence imaging to measure the position of polar kinetochore pairs relative to the spindle pole and astral microtubules (Fig. 4A,B). Kinetochores were on average 1.29 µm (±0.43 µm) from the pole in CENP-E-depleted cells (Fig. 4B,C; *n* = 2 159 kinetochores). In contrast, kinetochore pairs in CENP-Q-depleted cells were further from the pole, occupying an average distance of 2.62 µm (±0.99 µm) (Fig. 4B,C; *n* = 2, 107 kinetochores). Thus supporting our prediction that depletion of CENP-Q and CENP-E differentially affects the position of polar chromosomes relative to the pole.

To further validate this finding, we measured the distance of individual kinetochores to the spindle pole in monopolar spindles following depletion of CENP-E or CENP-Q. To generate monopolar spindles, cells were treated with monastrol, an inhibitor of Eg5 (also known as KIF11), for 90 min, which prevents centrosome separation in prophase (Mayer et al., 1999). This induces a high frequency of syntelic attachments (Lampson et al., 2004), enabling the assessment of positional changes in end-on attached kinetochores. Cells were then fixed and stained with antibodies against CENP-A (kinetochore marker) and γ-tubulin (spindle pole marker). The fixed cells were imaged, and the centre of mass for the monopole was identified based on the γ-tubulin signal (see Materials and Methods for details). We then automatically found the kinetochore positions based on the CENP-A staining and calculated the Euclidian distance to the monopole (Fig. 4D). The kinetochore-to-pole distances for each experiment (*n*≥4 independent experiments, ≥8 cells per experiment per condition) were then used to calculate the cumulative distribution frequency of the kinetochore-to-pole distances, the average of these cumulative distribution frequencies was then plotted (Fig. 4E,F). These plots show that depletion of CENP-E resulted in the distribution of kinetochore-to-pole distances moving closer to the monopole compared with that of control cells (Fig. 4E). We confirmed this result by treating cells with 300 nM GSK925293 to inhibit CENP-E motor activity (Fig. 4E). Conversely, kinetochores in CENP-Q-depleted cells (which also lack the anti-poleward CENP-E force due to its unbinding from kinetochores) occupied a similar mean distance between the monopole and kinetochores in control-siRNA-transfected cells, albeit with a much narrower standard deviation (Fig. 4F). Taken together, these data support our model that CENP-E slides kinetochores towards microtubule plus-ends, whereas CENP-Q-dependent mechanisms (that are independent of CENP-E recruitment) drive depolymerisation-coupled pulling that moves chromosomes towards or away from the pole depending on the microtubule geometry.

### The roles of CENP-Q in chromosome congression are independent of the recruitment of Plk1 and CENP-O complex subunits

**Fig. 5.**
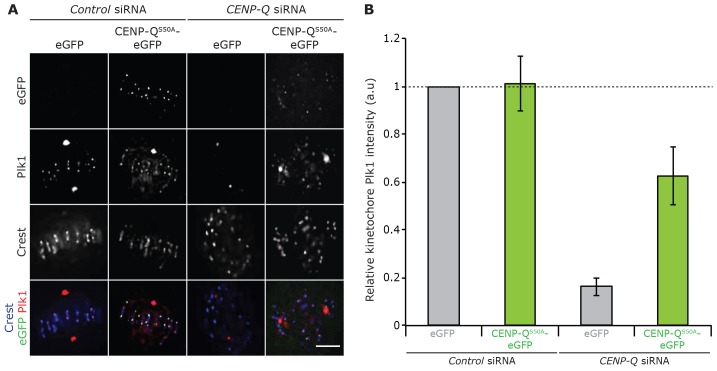
**CENP-Q^S50A^–eGFP rescues kinetochore recruitment of Plk1.** (A) Immunofluorescence microscopy images (*z*-slice) of a CENP-Q^S50A^–eGFP siRNA rescue experiment in HeLa K cells. Cells were treated with CENP-Q siRNA or control siRNA for 12 h and then transfected with an siRNA-resistant plasmid expressing CENP-Q^S50A^–eGFP or a control eGFP expression plasmid for a further 48 h. To reduce the effect of the mitotic stage on alignment, cells were arrested in metaphase with 1 µM MG132 for 90 min before fixation. Cells were stained with CREST antisera (blue) and an antibody against Plk1 (red). Scale bar: 5 µm. (B) Quantification of Plk1 levels in the CENP-Q^S50A^–eGFP siRNA rescue experiment. Staining intensities were determined at each kinetochore relative to that of CREST after background subtraction, *n* = 3, ≥300 kinetochores, 30 cells. The dashed line indicates CENP-Q levels in control-siRNA-treated cells. Error bars indicate ±s.d.

Previous reports have demonstrated that CENP-Q is phosphorylated by CENP-U-bound Plk1 *in vitro*, (Kang et al., 2011) and that Plk1 is required for localisation of CENP-E to the kinetochore (Ahonen et al., 2005; Nishino et al., 2006). Moreover, serine 50 in CENP-Q is phosphorylated *in vivo* (Rigbolt et al., 2011), suggesting that this might represent a key regulatory event. We therefore mutated serine 50 to alanine (CENP-Q^S50A^–eGFP) or to a phospho-mimicking aspartic acid residue (CENP-Q^S50D^–eGFP). We first tested whether the S50A mutant affected the recruitment of Plk1 to kinetochores. Consistent with our previous result (supplementary material Fig. S2), CENP-Q-depleted cells that had been transfected with an empty vector demonstrated an 84% reduction in Plk1 at kinetochores (±3.7%; Fig. 5A,B, *n* = 3, 300 kinetochores, 30 cells). In contrast, transfection with CENP-Q^S50A^–eGFP rescued kinetochore Plk1 levels to 62% (±12%; Fig. 5A,B, *n* = 3, 600 kinetochores, 60 cells). Previous work has shown that recruitment of Plk1 to kinetochores is dependent upon CENP-U (Kang et al., 2006). The presence of Plk1 and CENP-Q therefore indicates that CENP-U remains kinetochore bound, indicating that the CENP-O complex had successfully assembled at the kinetochore. Moreover, serine 50 is not reported as a Plk1 phosphorylation site (Santamaria et al., 2011); therefore, CENP-Q^S50A^–eGFP enables the separation of the role of CENP-Q in chromosome congression, in CENP-O complex assembly and in Plk1 recruitment.

**Fig. 6.**
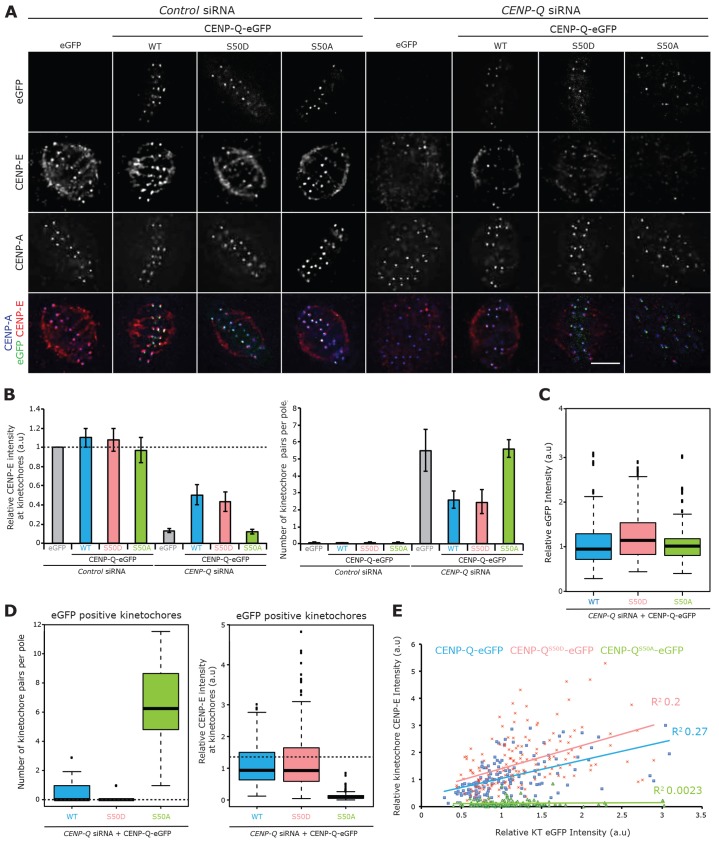
**CENP-Q serine 50 is required for CENP-E recruitment and orderly congression.** (A) Immunofluorescence microscopy images (*z*-slice) of CENP-Q^S50A^–eGFP and CENP-Q^S50D^–eGFP siRNA rescue experiments in HeLa K cells. Cells were treated with CENP-Q siRNA or control siRNA for 12 h and then transfected with an siRNA-resistant plasmid expressing CENP-Q–eGFP, CENP-Q^S50A^–eGFP or CENP-Q^S50D^–eGFP or a control eGFP expression plasmid for a further 48 h. To reduce the effect of the mitotic stage on alignment, cells were arrested in metaphase with 1 µM MG132 for 90 min before fixation. Cells were stained with antibodies against CENP-A (blue) and CENP-E (red). Scale bar: 5 µm. (B) Left panel, quantification of CENP-E levels in the CENP-Q^S50A^–eGFP and CENP-Q^S50D^–eGFP siRNA rescue experiments. Intensities were determined at each kinetochore relative to that of CENP-A after background subtraction, *n* = 3, ≥300 kinetochores, ≥30 cells. The dashed line indicates CENP-E levels in control-siRNA-treated cells. Error bars indicate ±s.d. Right panel, quantification of the average number of kinetochore pairs per pole in cells expressing CENP-Q^S50A^–eGFP and CENP-Q^S50D^–eGFP from three independent rescue experiments (120 poles per condition, 60 cells). (C) Box plot showing quantification of kinetochore transgene levels for each CENP-Q variant, *n* = 3, ≥150 kinetochores. The line represents the median, the box represents the interquartile range, whiskers represent the maximum values excluding outliers, dots represent outliers. (D) Analysis of cells with transgene-positive kinetochores. Box plots showing quantification of the average number of kinetochore pairs per pole, *n* = 3, ≥60 poles (left panel) and quantification of kinetochore CENP-E levels, *n* = 3, ≥150 (right panel). Dotted lines indicate the levels in control cells (taken from Fig. 6B). The line represents the median, the box represents the interquartile range, whiskers represent the maximum values excluding outliers, dots represent outliers. (E) A plot demonstrating the relationship between the kinetochore eGFP signal in cells rescued with CENP-Q–eGFP, CENP-Q–eGFP^S50A^ or CENP-Q–eGFP^S50D^ and the kinetochore CENP-E signal, *n* = 3, ≥150 kinetochores.

Next we investigated whether CENP-Q^S50A^–eGFP could rescue the CENP-Q depletion phenotype. Transfection with CENP-Q^S50A^–eGFP failed to rescue CENP-E recruitment when compared with that of control cells transfected with an empty vector (Fig. 6A,B, *n* = 3, 600 kinetochores, 60 cells). These cells still had an alignment defect, with approximately six kinetochore pairs trapped at each spindle pole (Fig. 6A,B, *n* = 3, 120 poles, 60 cells). To further validate the potential importance of phosphorylation at serine 50, we transfected CENP-Q-depleted cells with the phospho-mimetic CENP-Q^S50D^–eGFP transgene. This partially rescued CENP-E recruitment to 43% (±10%), comparable with that of wild-type CENP-Q–eGFP (Fig. 6A,B, *n* = 3, 600 kinetochores, 60 cells). These cells also displayed a less severe congression phenotype, having on average 2.4 (±0.8) kinetochore pairs per pole (Fig. 6A,B, *n* = 3, 120 poles, 60 cells).

Importantly, both CENP-Q^S50A^–eGFP and CENP-Q^S50D^–eGFP proteins were recruited to kinetochores (Fig. 6A). However, owing to the transient transfection, some cells had a low or non-detectable eGFP signal on kinetochores. We thus performed additional experiments and restricted our analysis to cells where a CENP-Q–eGFP, CENP-Q^S50A^–eGFP or CENP-Q^S50D^–eGFP signal could be detected on kinetochores. We first quantified this signal and confirmed that the levels were equivalent for the wild-type and mutant CENP-Q transgenes (Fig. 6C, *n*≥150 kinetochores per condition). Next, we counted the number of kinetochore pairs per pole in these cells and found that the CENP-Q–eGFP and CENP-Q^S50D^–eGFP transgenes, but not the CENP-Q^S50A^–eGFP transgene, almost completely rescued the phenotype (the median number of kinetochore pairs per pole was zero for the wild-type and S50D mutant constructs, compared with 6.5 for S50A; Fig. 6D, *n*≥150 kinetochores per condition). Likewise, we found that the CENP-Q–eGFP and CENP-Q^S50D^–eGFP transgenes restored the binding of CENP-E to kinetochores to ∼75% of the levels measured in control cells (Fig. 6D; *n*≥150 kinetochores per condition). Thus, the partial (∼50%) rescue in our population-based rescue experiments (see Fig. 6A,B) are simply a consequence of some cells not expressing the transgene. From these data, we also plotted the level of the transgene and that of CENP-E on individual kinetochores. This revealed a positive correlation for CENP-Q–eGFP and CENP-Q^S50D^–eGFP, indicating that the recruitment of CENP-E molecules is dependent upon the number of CENP-Q molecules at each kinetochore. As expected, this relationship was lost upon transfection with CENP-Q^S50A^–eGFP, with no CENP-E recruitment observed at high transgene levels (Fig. 6E, *n*≥150 kinetochores per condition). Taken together, these data support a role for CENP-Q in CENP-E loading onto kinetochores and depolymerisation-coupled pulling that is independent of Plk1 and the CENP-O complex.

## DISCUSSION

**Fig. 7.**
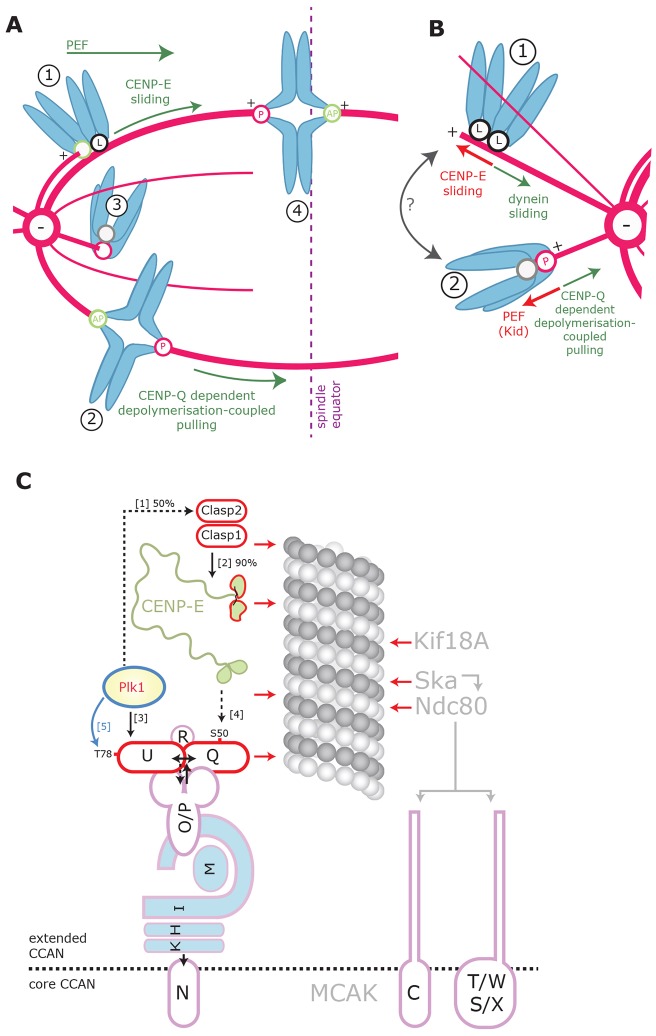
**A model for chromosome congression through the combined action of CENP-Q and CENP-E.** (A) Schematic showing a proposed model of chromosome congression for unaligned chromosomes positioned between the plate and the pole through the combined action of CENP-Q- and CENP-E-dependent mechanisms. Chromosome 1 is mono-orientated and laterally attached ‘L’ at the black sister kinetochore through CENP-E, this chromosome is able to congress to the plate through lateral sliding, driven by CENP-E, where it is then able to biorientate (as reported in Kapoor et al., 2006). Chromosome 2 is biorientated and able to congress to the metaphase plate by making persistent plateward movements that are driven by CENP-Q-dependent microtubule depolymerisation-coupled pulling at the poleward (P) kinetochore. Chromosome 3 is mono-orientated and will engage its free kinetochore with either the microtubule lattice or the plus-end of microtubules emanating from the opposite pole in order to congress through CENP-E- and/or CENP-Q-dependent pathways. Chromosome 4 is biorientated and aligned at the metaphase plate. AP, away from pole movement. (B) Schematic showing the mechanisms acting on chromosomes positioned behind the spindle poles. Arrows indicate the direction of the generated forces. CENP-E lateral sliding and polar ejection forces (PEFs) move chromosomes anti-poleward, whereas CENP-Q-dependent depolymerisation-coupled pulling and dynein-driven lateral sliding move chromosomes poleward. Force balance amongst these mechanisms dictates the distance of a chromosome from the spindle pole, and kinetochores are probably able to cycle between these attachment states (grey arrow). (C) Schematic showing kinetochore loading and phosphorylation dependencies. Solid black lines represent direct loading dependencies (percentage values indicate the level of dependency). Black dashed lines represent loading dependencies, which are indirect (or have not been shown to be direct). Blue lines represent phosphorylation events, and red arrows represent proteins that have been shown to make direct contact with the microtubule. Percentage dependencies were obtained from the following references: [1] (Maia et al., 2012), [2], (Maffini et al., 2009), [3] (Kang et al., 2006), [4] this study and [5] (Kang et al., 2011). Dependencies within CCAN were obtained from several references (Foltz et al., 2006; McClelland et al., 2007; Okada et al., 2006). The separation of the CCAN into core and extended components is taken from Westhorpe and Straight (Westhorpe and Straight, 2013).

Recent studies in human cells indicate that the majority of chromosomes rapidly convert from lateral to amphitelic attachment (biorientation) at the spindle equator as a result of kinetochores being organised into a ring-like conformation following nuclear envelope breakdown (Magidson et al., 2011). However, cells must have mechanisms that ensure chromosomes that are close to the pole early in mitosis can congress and thereby allow accurate disjunction of sister chromatids in anaphase. Our data support a model in which there are at least two distinct mechanisms that drive the congression of such polar chromosomes (Fig. 7A): (1) the well-documented lateral sliding of (non-biorientated) kinetochores by CENP-E (Kapoor et al., 2006) (Fig. 7A, chromosome 1), and (2) a role for CENP-Q that is independent of CENP-E recruitment in the movement of biorientated sisters to the metaphase plate (this study; Fig. 7A, chromosome 2). This conclusion is based on our observation that biorientated kinetochores are still able to congress in the absence of CENP-E, but not CENP-Q (where CENP-E is also unbound). Importantly, it is the absence of CENP-E from kinetochores in CENP-Q-depleted cells that allows us to rule out lateral sliding as the mechanism of congression and reveals a role for CENP-Q in the movement of biorientated sisters.

These CENP-Q-dependent movements are analogous to those described by Skibbens and colleagues 20 years ago, in which biorientated kinetochores can undergo long-duration motion towards the spindle equator (Skibbens et al., 1993). This motion is a consequence of one sister (facing the metaphase plate) remaining in a poleward-moving state, while the other sister maintains an away from pole state. Sister kinetochores that lack CENP-Q appear unable to sustain their poleward or anti-poleward state, resulting in stalling and a failure to congress. An important question will be to understand how CENP-Q contributes to this process (see below).

How do cells initially move chromosomes to spindle poles? Existing models (see Kim et al., 2008; Skibbens et al., 1993) state that movement to the pole is necessary in order to increase the probability of biorientation due to the higher density of microtubules. However, our data show that CENP-E-dependent sliding of laterally attached kinetochores will move chromosomes that are behind the pole further away from the pole and towards the plus-end of astral-microtubules. At the same time, chromosome arms are pushed away from the pole by the polar ejection force (Stumpff et al., 2012). To counter these anti-poleward forces, dynein motors can slide laterally attached kinetochores towards the pole (Yang et al., 2007). We propose that there is a second CENP-Q-dependent process that moves chromosomes poleward. We suggest that this process is likely to reflect the effect of CENP-Q on mono-orientated kinetochores. If direct, this would mean a requirement for CENP-Q in depolymerisation-coupled pulling and poleward kinetochore motility. It follows that the failure of a biorientated sister pair to congress is therefore, most likely, also due to a defective poleward kinetochore movement (depolymerisation-coupled pulling), rather than an away from pole kinetochore (pushing) movement. It will be important to determine how kinetochores are converted between lateral, mono-orientated and biorientated states (chromosomes 1–4 in Fig. 7A, chromosomes 1 and 2 in Fig. 7B). In this regard, recent work has revealed that the transition from CENP-E-dependent lateral attachment to an end-on configuration requires the microtubule depolymerase MCAK (Shrestha and Draviam, 2013). MCAK is proposed to depolymerise the astral microtubule back to the kinetochore, thus promoting the mono-orientation of the kinetochore. Overall, current data suggests that kinetochores utilise poleward and anti-poleward mechanisms to position themselves at the optimum distance from the spindle pole to promote biorientation.

How could CENP-Q contribute to depolymerisation-coupled pulling? One possibility is that CENP-Q indirectly affects depolymerisation-coupled pulling by affecting the binding of other factors to kinetochores (see schematic in Fig. 7C). However, we can rule out the loss of the CENP-O complex or Plk1, which both remain kinetochore-bound in cells expressing the S50A mutant (which phenocopies CENP-Q depletion). In addition, our data show that CENP-E is not required for depolymerisation-coupled pulling (see above). Although it remains possible that an unknown protein is recruited to kinetochores in manner that is dependent on serine 50, we favour the alternative idea that CENP-Q mediates a direct effect on kinetochore–microtubule dynamics. Support for this idea comes from *in vitro* biochemistry, which demonstrates that the purified CENP-Q protein can directly bind to taxol-stabilised microtubules *in vitro* (Amaro et al., 2010). We do find that depletion of CENP-Q reduces the turnover of kinetochore microtubules *in vivo* (supplementary material Fig. S3). However, CENP-E depletion is reported to have the same effect (Maffini et al., 2009), while still allowing the congression of biorientated kinetochores through depolymerisation-coupled pulling (this study). Therefore, we cannot yet attribute these changes in kinetochore–microtubule dynamics to the observed defects in chromosome movement in CENP-Q-depleted cells.

Our data suggest that phosphoregulation of CENP-Q through serine 50 is an important regulatory step in controlling chromosome congression. An attractive idea is that this phosphorylation event allows the direct binding of CENP-E to CENP-Q. However, CENP-E remains bound to kinetochores in CENP-H- or CENP-L-depleted cells – CCAN proteins that are required for CENP-Q binding to kinetochores (Amaro et al., 2010; McClelland et al., 2007; Mchedlishvili et al., 2012). Moreover, binding of CENP-E to kinetochores in CENP-Q-depleted cells is partially rescued following depolymerisation of microtubules with nocodazole (supplementary material Fig. S4). Thus, a direct mechanism seems unlikely. An alternative possibility is that CENP-Q modulates kinetochore–microtubule dynamics in such a way that CENP-E can be recruited. As discussed above, these same changes in microtubule dynamics could also explain the defects in depolymerisation-coupled pulling. Finally, the kinase that is most likely to be responsible for the phosphorylation of serine 50 would be the CENP-U-bound pool of Plk1 (Kang et al., 2006). CENP-Q has been shown to be a substrate for Plk1 *in vitro* (Kang et al., 2011). However, stable isotope labelling by amino acids in cell culture (SILAC) experiments show that phosphorylation of serine 50 is not sensitive to the depletion or inhibition of Plk1 in human cells (Santamaria et al., 2011). Future work will clearly be required to identify the kinase responsible for the regulation of CENP-Q function.

## MATERIALS AND METHODS

### Cell culture, siRNA transfection and drug treatments

HeLa-E1 and HeLa K cells were grown in Dulbecco's modified Eagle's medium (DMEM) containing 10% foetal calf serum, 100 U/ml penicillin and 100 µg ml^−1^ streptomycin at 37°C under 5% CO_2_ in a humidified incubator. The Histone2B–mRFP cell line was maintained in 500 µg ml^−1^ G418 and the eGFP–CENP-A eGFP–centrin1 cell line was maintained in 500 µg ml^−1^ G418 and 0.3 µg ml^−1^ puromycin. All other cell lines were maintained in non-selective medium. siRNA oligonucleotides (53 nM) were transfected using oligofectamine (Invitrogen) for 48 h [24 h in modified Eagle's medium (MEM) then changed to DMEM for a further 24 h] according to the manufacturer's instructions. The siRNA oligonucleotide sequences used were control (Samora et al., 2011), CENP-Q (5′-GGUCUGGCAUUACUACAGGAAGAAA-3′ Stealth, Invitrogen), CENP-Q-2 (5′-CAGAGUUAAUGACUGGGAAUAUUCA-3′ Stealth, Invitrogen), CENP-P (Amaro et al., 2010) and CENP-E (5′-ACUCUUACUGCUCUCCAGUdTdT-3′, Ambion). Drug treatments were performed at the following concentrations and time periods – nocodazole (Tocris) 14 h at 1 µg ml^−1^, GSK923295 (CENP-E inhibitor; Haoyuan Chemexpress) 14 h at 300 nM, taxol (Tocris) at 10 µM for 60 min, monastrol (Tocris) at 1 µM for 90 min and MG132 at 1 µM for 90 min.

### Molecular biology and siRNA rescue experiments

To generate a human CENP-Q–eGFP expression vector, the CENP-Q coding sequence was amplified by using PCR (using primers MC246 and MC248) and ligated into pEGFP-N1 (empty vector; Clontech) using *Bam*HI and *Eco*RI (pMC276). pMC276 was then mutated using a quick change site-directed mutagenesis kit (Stratagene) and the primers MC297 \(5′-GACAAAGCTAATGAAGAAGGCCTAGCGTTGCTCCAAGAGGAAATAGATAAAATGGTAGAG-3′) and MC298 (5′-CTCTACCATTTTATCTATTTCCTCTTGGAGC AACGCTAGGCCTTCTTCATTAGCTTTGTC-3′), to render the transgene resistant to the CENP-Q siRNA oligonucleotide (pMC308). This siRNA-resistant CENP-Q construct was then used as a template for the generation of CENP-Q phospho-mutants using a quick change site-directed mutagenesis kit (Stratagene) and the following primers: CENP-Q^S50A^-eGFP (5′-ATAAAAATCATCTGAAAGATCTGGCTTCTGAAGGACAAACAAAGCAC-3′) and (5′-GTGCTTTGTTTGTCCTTCAGAAGCCAGATCTTTCAGATGATTTTTAT-3′), CENP-Q^S50D^-eGFP (5′-AAAAAATAAAAATCATCTGAAAGATCTGGATTCTGAAGGACAAACAAAGCACACTAAC-3′) and (5′-GTTAGTGTGCTTTGTTTGTCCTTCAGAATCCAGATCTTTCAGATGATTTTTATTTTTT-3′). For siRNA rescue experiments, cells were transfected with CENP-Q or control siRNAs and grown for 14 h in MEM. The medium was then changed to DMEM and the cells transfected with 1 µg of pEGFP-N1 (empty vector; Clontech) or a CENP-Q transgene using FuGene6 transfection reagent (Roche); cells were then incubated for a further 48 h before analysis.

### Immunofluorescence microscopy

Cells were fixed at room temperature for 10 min in 20 mM PIPES pH 6.8 containing 10 mM EGTA, 1 mM MgCl_2_, 0.2% Triton X-100 and 4% formaldehyde. Cells were then were then washed with PBS every 10 min three times before blocking with 3% BSA in PBS for 30 min. After blocking, the fixed cells were incubated for 1 h with primary antibodies: rabbit anti-CENP-O (1∶500; McAinsh et al., 2006), mouse anti-CENP-A (1∶500; Abcam), mouse anti-α-tubulin (1∶1000; Sigma-Aldrich), CREST antisera (1∶250; Antibodies Incorporated), rabbit anti-CENP-E [1∶1,500; (Meraldi et al., 2004)], rabbit anti-Ska3 (1∶250) (a kind gift from Anna Santamaria, University of Basel, Switzerland), rabbit anti-CENP-Q (1∶250; Rockland), rabbit anti-Plk1 (1∶250; Santa Cruz), rabbit anti-γ-tubulin (1∶200; Abcam), mouse monoclonal anti-Ndc80/Hec1 (1∶1000; Abcam). Cells were then washed with PBS every 10 min three times and incubated for 30 min with AlexaFluor-conjugated highly cross-adsorbed secondary antibodies raised in goat (Invitrogen). Three-dimensional image stacks of mitotic cells were acquired in 0.2 µm steps using a 100× oil-immersion 1.4 NA objective lens on an Olympus DeltaVision Elite microscope (Applied Precision, LLC) equipped with a DAPI, fluorescein isothiocyanate (FITC), Rhodamine or Texas Red, CY5 filter set (Chroma), solid state light source and a CoolSNAP HQ camera (Roper Scientific). Image stacks were deconvolved using SoftWorx (Applied Precision, LLC), and figures were generated with Photoshop and Illustrator (Adobe). Fluorescence-intensity measurements were made manually using SoftWorx, subtracting background values and normalising to the intensity of CENP-A or CREST on the same kinetochore.

### Live-cell imaging

Fluorescence time-lapse imaging of cells co-expressing H2B–eGFP and mRFP–α-tubulin was performed on a Personal Deltavision microscope (Applied Precision, LLC), using a 40× NA 1.3 objective, with GFP (excitation 475/28, emission 525/50) and mCherry (excitation 575/25, emission 632/60) filter set, Quad-mCherry dichroic mirror (reflection bands 381–401, 464–492, 561–590, 625–644, transmission bands 409–456, 500–553, 598–617, 652–700) (Chroma), Xenon light source and a CoolSNAP HQ2 camera (Roper Scientific). Image stacks (7×2 µm *z*-sections) were collected every 3 min for a total time of 10 h.

### Immunoblotting

Whole-cell protein extracts were prepared using a liquid nitrogen grinding technique as previously described (McClelland et al., 2007). Immunoblotting was performed as described previously (McAinsh et al., 2006). Primary mouse anti-α-tubulin (1∶10,000; Sigma-Aldrich) or rabbit anti-CENP-Q (1∶500; Rockland), and secondary anti-mouse or anti-rabbit horseradish-peroxidase-conjugated secondary antibodies (1∶10,000; Amersham) were diluted in PBS 0.1% Tween20 with 3% milk powder (Marvel). Primary antibodies were incubated for 14 h overnight at 4°C and secondary antibodies for 1 h at room temperature.

## Supplementary Material

Supplementary Material
