# Supplementary material for: Chromosome congression is promoted by CENP-Q- and CENP-E-dependent pathways
Source: J Cell Sci. 2015 Jan 1;128(1):171–84. doi: 10.1242/jcs.163659 (PMC4282051; doi:10.1242/jcs.163659)
Supplement: Supplementary Material [file supp_128_1_171__index.html]

Chromosome congression is promoted by CENP-Q- and CENP-E-dependent pathways — Supplementary Material 

# Chromosome congression is promoted by CENP-Q- and CENP-E-dependent pathways

## JCS163659 Supplementary Material

**Files in this Data Supplement:**

- **Supplementary Material**
