# Supplementary material for: Chromosome congression is promoted by CENP-Q- and CENP-E-dependent pathways
Source: J Cell Sci. 2015 Jan 1;128(1):171–84. doi: 10.1242/jcs.163659 (PMC4282051; doi:10.1242/jcs.163659)
Supplement: Supplementary Material [file supp_128.1.171_JCS163659.pdf]

Supplementary Figure 1

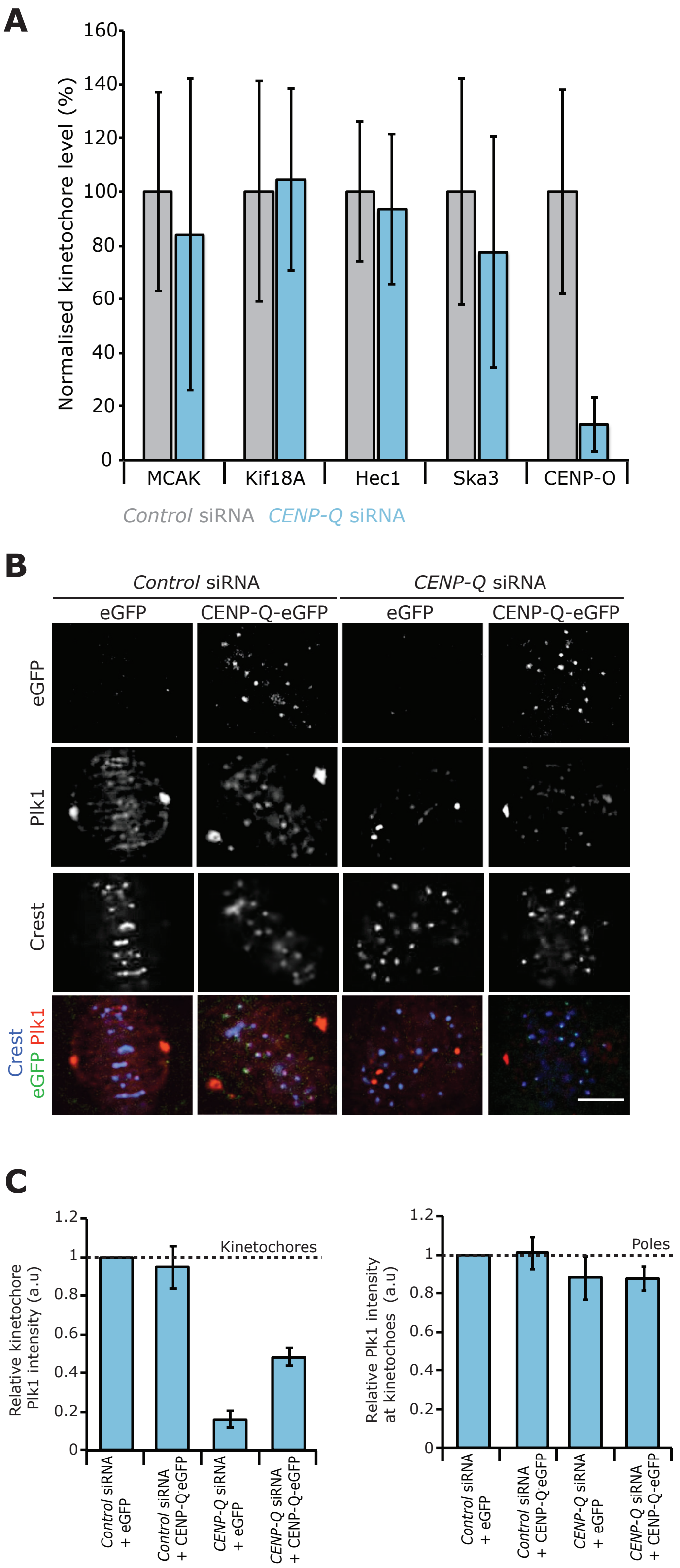

Supplementary Figure 2

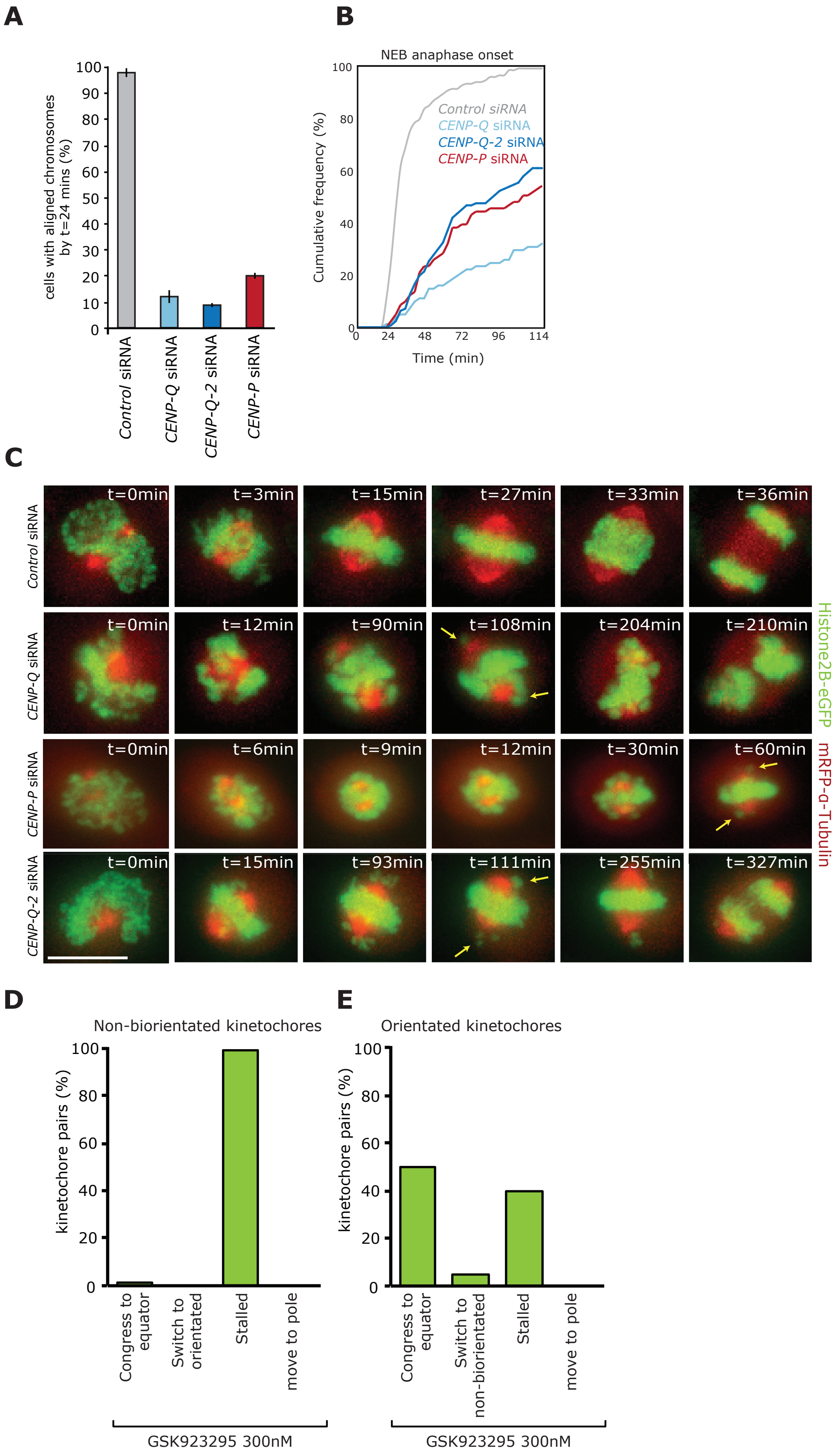

Supplementary Figure 3

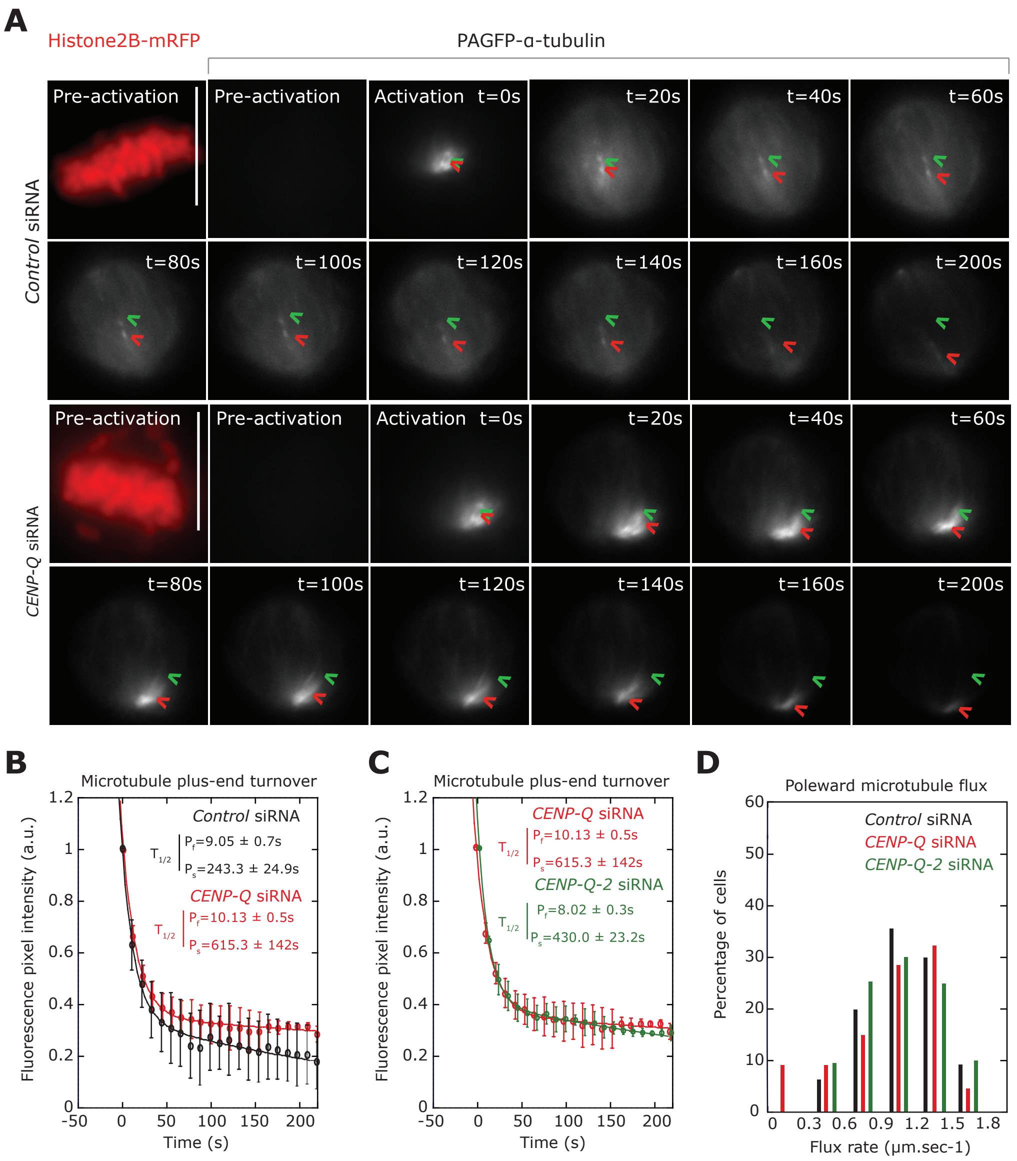

Supplementary Figure 4

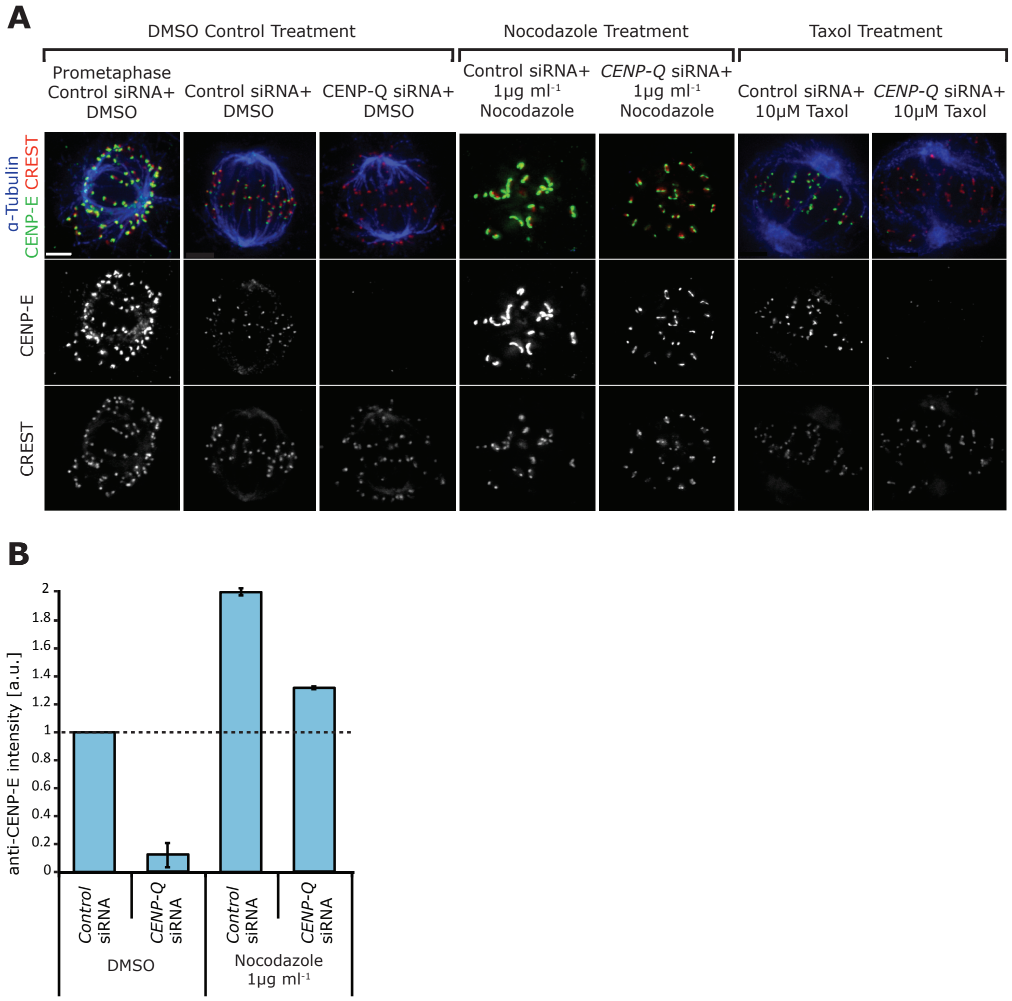

Supplementary Figure Legends

Supplementary Figure 1 – Kinetochore-binding of Plk1, Ndc80, Ska, CENP-O, Kif18A and MCAK in CENP-Q depleted cells.

(A) Quantification of Hec1, Ska3, CENP-O, Kif18A and MCAK levels at congressed kinetochore pairs in CENP-Q depleted metaphase HeLa E1 cells, n=2 ≥150 kinetochores per condition.

(B) Immunofluorescence images of Plk1 rescue experiment in HeLa K cells. Cells were treated with control or CENP-Q siRNA for 12 hr and then transfected with an siRNA resistant plasmid expressing CENP-Q-eGFP or a control eGFP expression plasmid for a further 48 hr. To reduce the effect of mitotic stage on alignment cells were arrested in metaphase with 0.1μM MG132 for 90 min prior to fixation, and stained with antibodies against Crest (blue) and Plk1 (red). Scale bar indicates 5μm.

(C) Left panel: Quantification of kinetochore Plk1 levels in the CENP-Q rescue experiment, n=3, ≥300 kinetochores per condition. Right panel: Quantification of polar Plk1 signal in the CENP-Q rescue experiment. A 5×5 pixel box was used to measure the mean intensity at the center of the pole, n=3, 120 poles per condition.

Supplementary Figure 2 – CENP-Q congression phenotypes can be recreated using a second CENP-Q siRNA, CENP-P siRNA and CENP-E inhibition

(a) Graph showing the percentage of HeLa E1 cells expressing histone-2B-eGFP and mRFP-α-tubulin cells to have aligned chromosomes at 24 minutes after nuclear envelope breakdown when treated with CENP-Q (light blue), CENP-Q-2 (dark blue) and control (grey) siRNA for 48 hr or CENP-P (red) siRNA for 72 hr.

(b) Quantification of nuclear envelope breakdown (NEB) to anaphase onset time in HeLa E1 cells stably expressing H2B–eGFP/mRFP–α-tubulin. Cells were treated with CENP-Q (light blue), CENP-Q-2 (dark blue) and control (grey) siRNA for 48 hr or CENP-P (red) siRNA for 72 hr.

(c) Frames from live-cell movies of HeLa E1 cells expressing H2B–eGFP/mRFP–α-tubulin. Cells were treated with control (top row) CENP-Q (second row) CENP-Q-2 (fourth row) siRNA for 48 hr and CENP-P siRNA (third row) for 72 hr. Yellow arrows point to unaligned kinetochores. Scale bar indicates 10μm.

(d) The fates of non-biorientated kinetochore pairs over 5 min in HeLa K cells expressing eGFP-CENP-A and eGFP-Centrin1 after 14-hr treatment with the CENP-E inhibitor 300 nM GSK923295.
(e) The fates of biorientated pairs over 5 min in HeLa K cells expressing eGFP-CENP-A and eGFP-Centrin1 after 14-hr treatment with the CENP-E inhibitor 300nM GSK923295.

Supplementary Figure 3 – Depletion of CENP-Q decreases kinetochore microtubule fibre plus end turnover.
(a) Frames from live-cell movies before and after photoactivation of stable photoactivatable GFP (PAGFP)–α-tubulin/H2B–mRFP HeLa cells treated with control or CENP-Q (and CENP-Q-2) siRNAs for 48 hr. Photoactivatable GFP–α-tubulin fluorescence was activated in a circular region near the chromosome mass in metaphase cells (detected by the H2B–mRFP signal). An H2B–mRFP (DNA) frame is shown for the first time point of the live-cell movie after activation. Scale bar represents 10 μm.
(b-c) Quantification of fluorescence intensity decay of the activated PAGFP- α-tubulin over time in control, CENP-Q (d) or CENP-Q-2 siRNA (e) treated cells. The lines through the data points (mean values) were fitted to a double exponential equation of the type $I = Pf \exp(-kft) + Ps \exp(-kst)$. Error bars indicate SD. The calculated half-lives of the fast (K-MT) and slow (non-K-MT) MT populations are indicated with the fitting error (SD).
(d) Quantification of poleward microtubule flux rates in cells treated with control (black bars), CENP-Q (red bars) or CENP-Q-2 siRNA (green bars) n = 20 cells for each condition.

Supplementary Figure 4 – CENP-E binding in CENP-Q depleted cells is partially rescued by depolymerisation of microtubules
(a) Immunofluorescence microscopy images of HeLa E1 cells stained with CENP-E (green), CREST (red) and α-tubulin (blue) antibodies. Images are a z-projection (10 focal planes at 0.2 μm spacing). Cells were treated with control siRNA or CENP-Q siRNA for 48 hr and either DMSO (14 hr), nocodazole (1 μM, 14 hr) or taxol (10 μM, 1 hr) before fixation.

(b) Quantification of CENP-E immunofluorescence levels in HeLa E1 cells treated with control siRNA or CENP-Q siRNA for 48 hr and either DMSO (14 hr) or nocodazole (1 μM, 14 hr) before fixation. Intensities are determined at each kinetochore relative to CREST and with background subtraction, n≥100 kinetochores per condition from 2 independent experiments. Dashed line indicates CENP-E levels in control siRNA cells + DMSO. Error bars indicate SD.

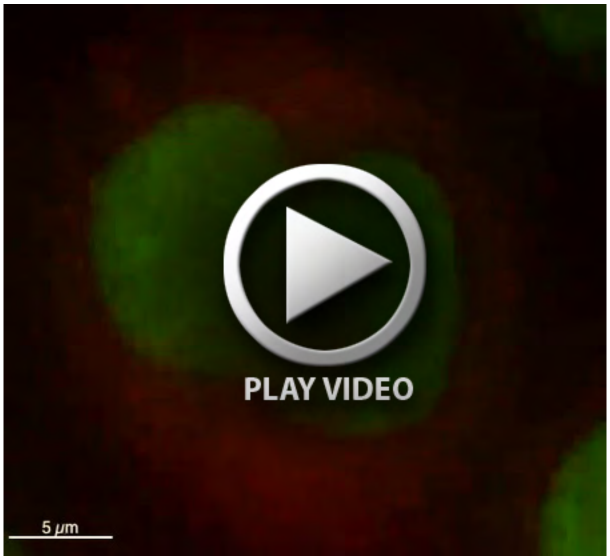

Movie 1. Control siRNA treated HeLa histone-2B-GFP α-tubulin-mRFP cell

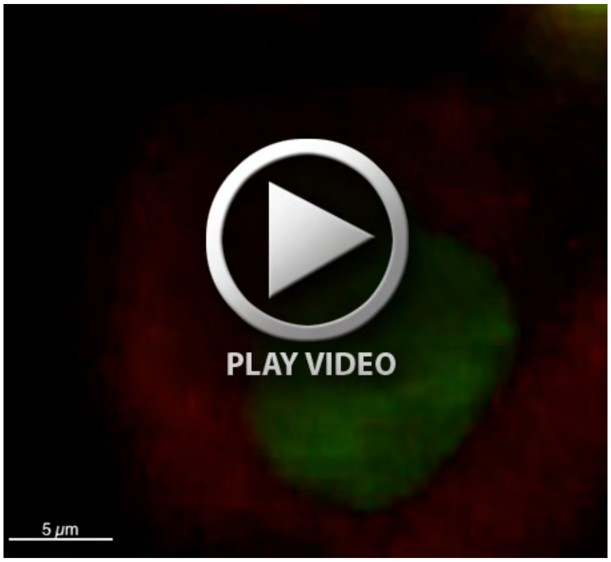

Movie 2. CENP-Q siRNA treated HeLa histone-2B-GFP α-tubulin-mRFP cell

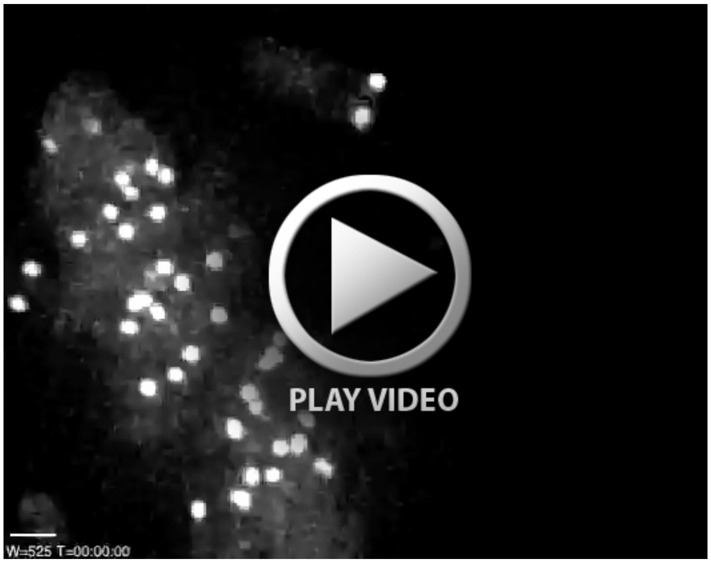

Movie 3. A CENP-Q depleted kinetochore pair switching from an orientated to non-biorientated state

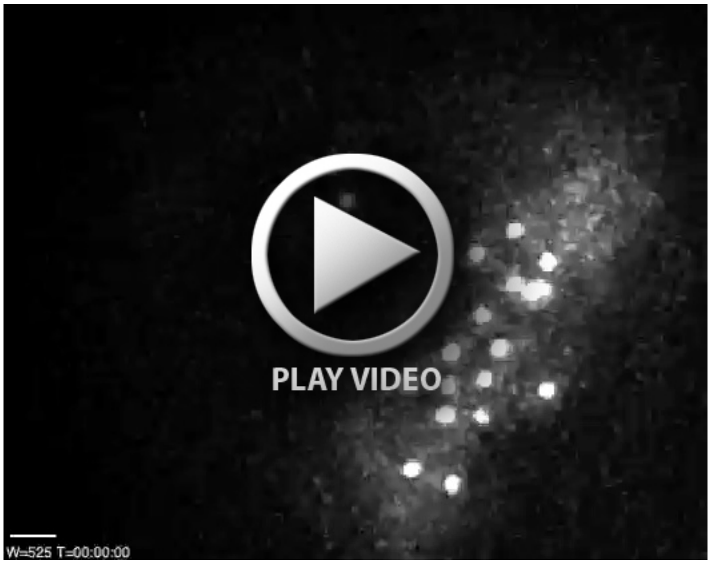

Movie 4. An orientated stalled kinetochore pair during CENP-Q depletion

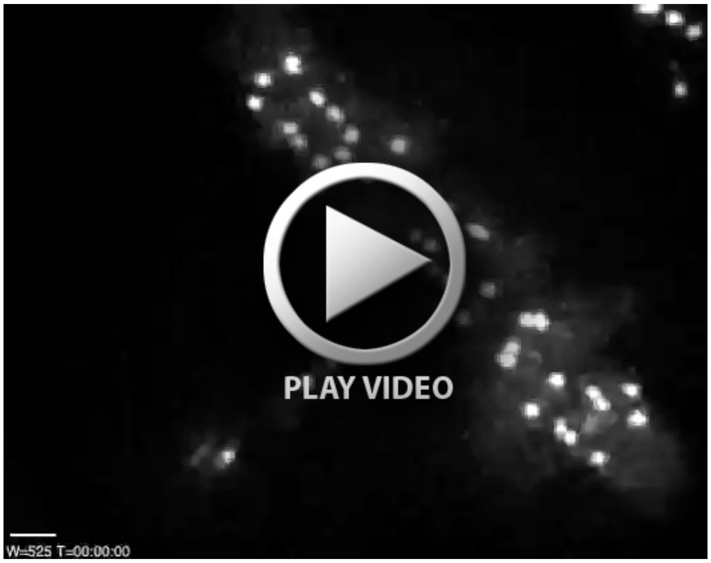

Movie 5. An orientated congressing kinetochore pair during CENP-E depletion

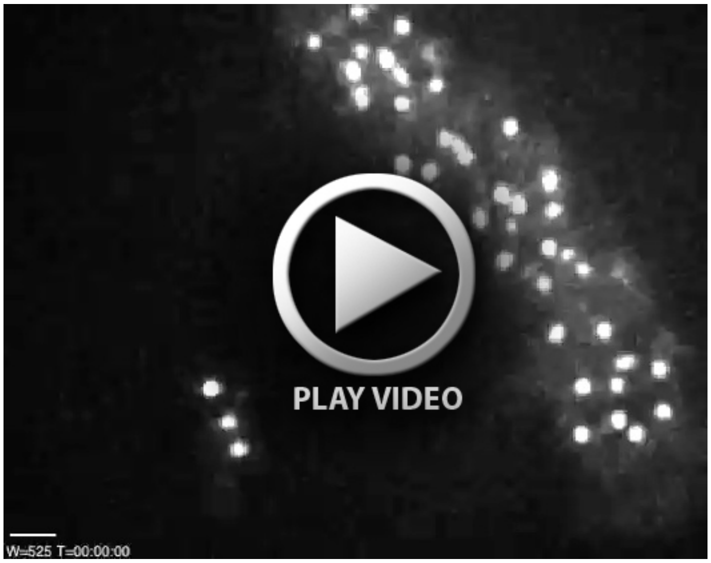

Movie 6. A non-biorientated stalled kinetochore pair during CENP-E depletion
